# Activation of an AMP-activated protein kinase is involved in post-diapause development of *Artemia franciscana *encysted embryos

**DOI:** 10.1186/1471-213X-9-21

**Published:** 2009-03-16

**Authors:** Xiao-Jing Zhu, Jie-Qiong Dai, Xin Tan, Yang Zhao, Wei-Jun Yang

**Affiliations:** 1 Institute of Cell Biology and Genetics, College of Life Sciences, Zijingang Campus, Zhejiang University, Hangzhou, Zhejiang 310058, PR China; 2 State Conservation Center for Gene Resources of Wildlife, Key Laboratory of Conservation Genetics and Reproductive Biology for Wild Animals of the Ministry of Education, Hangzhou, Zhejiang 310058, PR China; 3 College of Life Sciences, Zijingang Campus, Zhejiang University, Hangzhou, Zhejiang 310058, PR China

## Abstract

**Background:**

Cysts of *Artemia *can remain in a dormant state for long periods with a very low metabolic rate, and only resume their development with the approach of favorable conditions. The post-diapause development is a very complicated process involving a variety of metabolic and biochemical events. However, the intrinsic mechanisms that regulate this process are unclear.

**Results:**

Herein we report the specific activation of an AMP-activated protein kinase (AMPK) in the post-diapause developmental process of *Artemia*. Using a phospho-AMPKα antibody, AMPK was shown to be phosphorylated in the post-diapause developmental process. Results of kinase assay analysis showed that this phosphorylation is essential for AMPK activation. Using whole-mount immunohistochemistry, phosphorylated AMPK was shown to be predominantly located in the ectoderm of the early developed embryos in a ring shape; however, the location and shape of the activation region changed as development proceeded. Additionally, Western blotting analysis on different portions of the cyst extracts showed that phosphorylated AMPKα localized to the nuclei and this location was not affected by intracellular pH. Confocal microscopy analysis of immunofluorescent stained cyst nuclei further showed that AMPKα localized to the nuclei when activated. Moreover, cellular AMP, ADP, and ATP levels in developing cysts were determined by HPLC, and the results showed that the activation of *Artemia *AMPK may not be associated with cellular AMP:ATP ratios, suggesting other pathways for regulation of *Artemia *AMPK activity.

**Conclusion:**

Together, we report evidence demonstrating the activation of AMPK in *Artemia *developing cysts and present an argument for its role in the development-related gene expression and energy control in certain cells during post-diapause development of *Artemia*.

## Background

*Artemia *is a species of primitive crustaceans capable of producing diapause encysted embryos (cysts) to survive adverse conditions. The cyst, composed of about 4000 cells and developmentally arrested at the gastrula stage, is remarkably resistant to physiologic stressors [1]. Diapause embryos remain in dormancy and will not resume development until they are activated by transient exposure to a specific environmental stimulus. Activated cysts require only suitable environmental conditions to resume metabolism and development, and eventually emerge as fully formed nauplii [2]. The special pattern of the cyst development mades it an ideal system for biological study.

Thus far, the sequence of events accompanying the diapause and resumption of development in *Artemia *has been investigated extensively. Two proteins, p26 and artemin, are present in large amounts in encysted embryos. p26 exhibits reversible nuclear-cytoplasmic translocation and plays an important role as a molecular chaperone, while artemin is a RNA-binding protein with high thermal stability and may act as a RNA chaperone [1,3-5]. Previous studies have also suggested intracellular pH (pHi) as a key cellular signal in the metabolic and developmental switching [6,7]. Interestingly, post-diapause development takes place in the absence of DNA synthesis and cell division [8,9], and is known to be a very complicated process involving a variety of metabolic events. These events include the catabolism of trehalose, degradation of yolk platelets, protein synthesis, gene transcription, and other events, coupled with a large number of energy changes [10-14]. However, the intrinsic mechanisms of this complicated process remain unclear.

Adenosine monophosphate-activated protein kinase (AMPK) is a cellular energy sensor that is conserved throughout eukaryotes. AMPK also plays an important role in the control of the whole body's energy balance [15,16]. AMPK homologues exist as heterotrimeric complexes consisting of a catalytic α-subunit and non-catalytic β- and γ-subunits [16]. Activation of AMPK absolutely requires phosphorylation at a specific threonine residue (Thr-172) of the α-subunit by upstream kinases (LKB1 or CaMKKβ), and allosterically by increases in the AMP:ATP ratio [17,18]. AMPK activation may also be elicited by other cellular signals, such as glycogen [19].

The upstream kinase, LKB1, signals through AMPK to regulate multiple metabolic processes. There is also evidence that AMPK has a more complex role in the regulation of diverse cellular processes, including the cell cycle, proliferation, and others through the LKB1→AMPK pathway [15]. The differential tissue-specific and subcellular localization of AMPK is critical in investigating its functions. In general, nuclear AMPK activation may elicit long-term changes in gene expression, whereas cytosolic AMPK may function in the modulation of more immediate metabolic and homeostatic responses [19].

The main objective of this study was to explore the role of AMPK in the *Artemia *life cycle. In a previous study, we have identified an *Artemia *AMPK gene (*Afr-AMPKα1*) which is differentially expressed during the developmental stages [20]. Herein we report the activation of AMPK in *Artemia *post-diapause developmental stages and discuss its possible roles in this process.

## Results

### AMPK activation during the post-diapause developmental stages of *Artemia*

In our previous study, an *Artemia *AMPKα gene (*Afr-AMPKalpha1*) was isolated and the pattern of the gene expression was studied [20]. The deduced amino acid sequence near threonine (172) in the T-loop region of this protein is identical with human AMPKα. Thus, a phospho-AMPKα (Thr172) antibody designed corresponding to the residues surrounding Thr172 of human AMPKα and widely used in detecting activated AMPK in various organisms was used in the present study.

**Figure 1:**
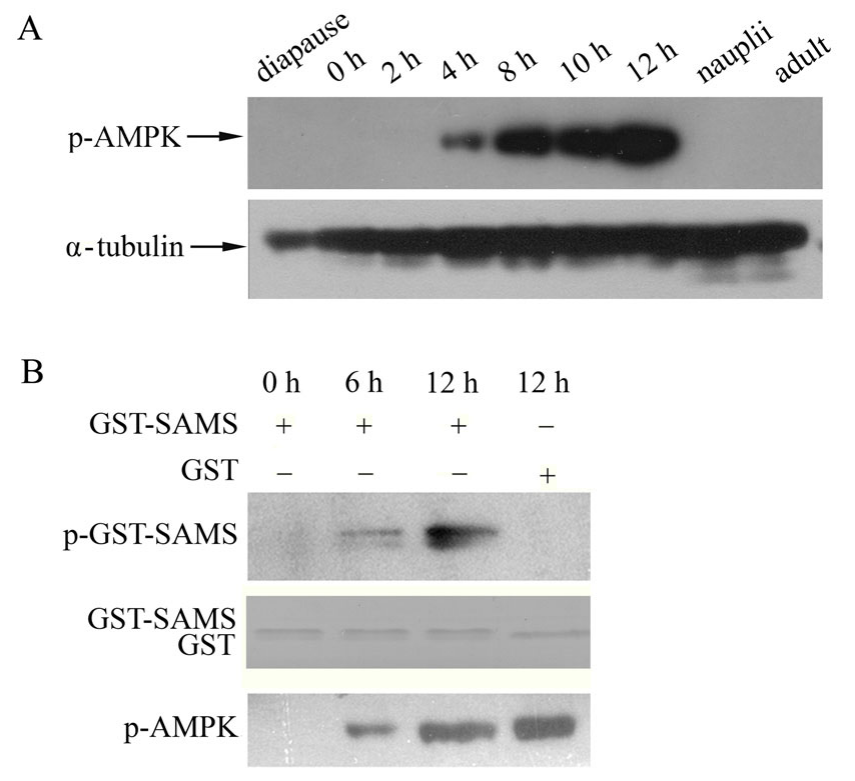
**Activation of AMPK during developmental stages of *Artemia***. A. phosphorylated AMPK was detected using phospho-AMPK (Thr172) antibody by immunoblotting extracts from diapause embryos, developing post-diapause embryos (0 – 12 h), nauplii, and adults. α-Tubulin was used as a loading control. B. *In vitro *kinase assay to test AMPK activity from PEG-precipitated proteins. GST-SAMS or GST from the reaction buffer were separated by 15% SDS-PAGE and phosphorylated proteins were detected by Western blotting using Biotinylated Phos-tag™. Coomassie staining of GST proteins as a loading control is shown in the lower panel. The presence of phosphorylated AMPK in the precipitates was determined by Western blotting with phospho-AMPK antibody. p-AMPK, phosphorylated AMPK; p-GST-SAMS, phosphorylated GST-SAMS.

The phosphorylation of AMPK during *Artemia *developmental stages was analyzed by Western blotting. The results showed that AMPK is initially phosphorylated in 4-h incubated embryos (Figure 1A). Subsequently, the phospho-AMPK level increased as the post-diapause development proceeded. No signal of phospho-AMPK was detected in the free-swimming nauplius or adults (Figure 1A). The molecular weight of the detected protein is about 60 kDa, similar to the predicted molecular weight of Afr-AMPKALPHA1 [20].

The kinase activity of AMPK was assayed with a GST-fused AMPK-specific substrate, GST-SAMS [21]. The SAMS peptide is based on the sequence surrounding serine 79 within rat acetyl-coA carboxylase and has been used most frequently for AMPK activity determination. Purified GST-SAMS was incubated with PEG extracts from embryos at different developmental stages, and the phosphorylated GST-SAMS was detected by Western blotting. As shown in Figure 1B, no signal of phosphorylated GST-SAMS was detected in 0-h incubated embryos in which phospho-AMPK was undetectable. Furthermore, the level of phospho-GST-SAMS increased as the level of phospho-AMPK increased (Figure 1B, 6–10 h). These results suggest that the activity of AMPK was elevated when the level of phosphorylated AMPK increased (Figure 1B), indicating that *Artemia *AMPK, like AMPK in other organisms, is activated by phosphorylation.

**Figure 2:**
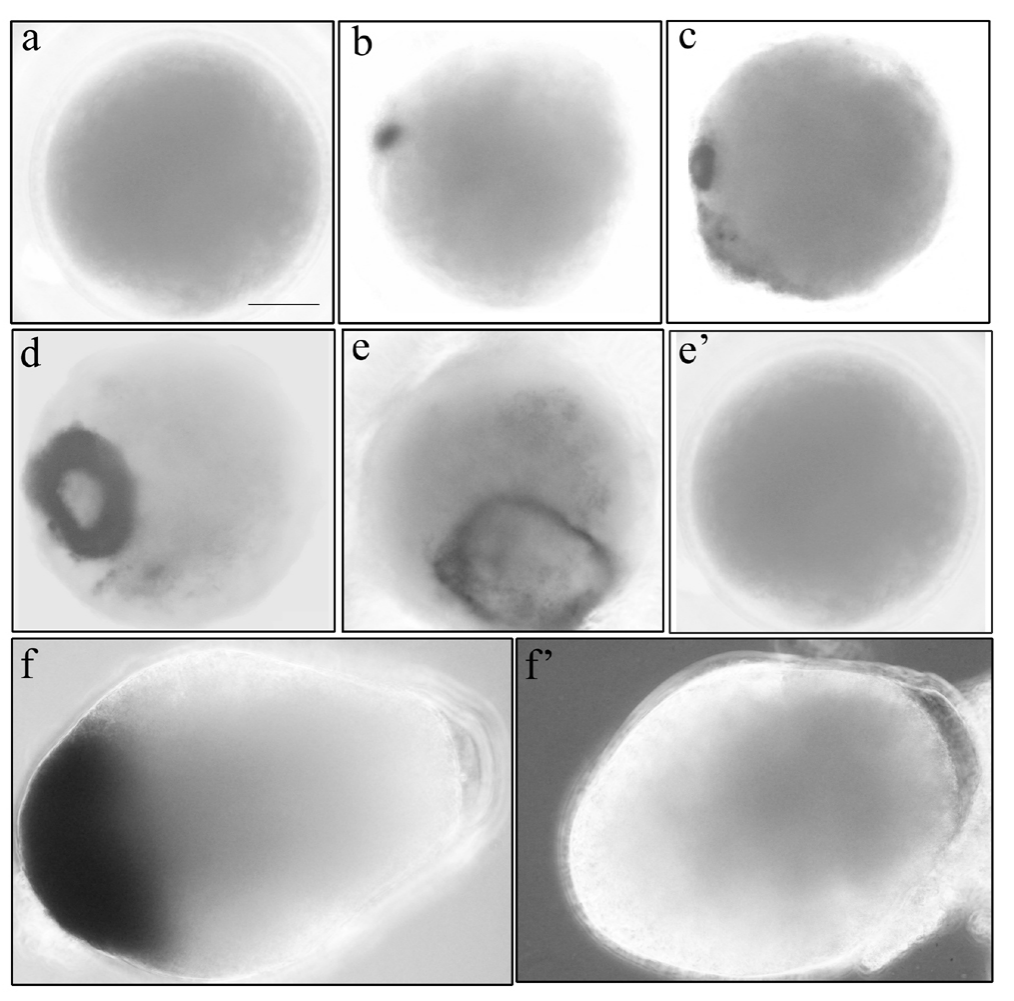
**Location of activated AMPK in developing cysts by whole mount immunohistochemistry**. Decapsulated embryos developing for 2, 4, 6, 8, 10, and 12 h were fixed in 4% paraformaldehyde, permeated by 0.1% Triton X-100, incubated with phospho-AMPK (Thr172) antibody (a – f), and subsequently detected with the AP-conjugated secondary antibody by NBT/BCIP staining. e' and f' represent control embryos that were not incubated with the primary antibody. Embryos are shown at the same magnification.

Using whole-mount immunohistochemistry, we have examined the distribution of activated AMPK in developing embryos (Figure 2). The results also illustrate that AMPK is activated in 4-h embryos and embryos at later stages. Activated AMPK was predominantly located in the ectoderm region of embryos at early stages in a ring shape (Figures 2b–d). As development proceeded, the activated loop enlarged (Figure 2e), and in late embryos that have a pear shape, AMPK was activated in the head region (Figure 2f).

### Nuclear translocation of AMPK

**Figure 3:**
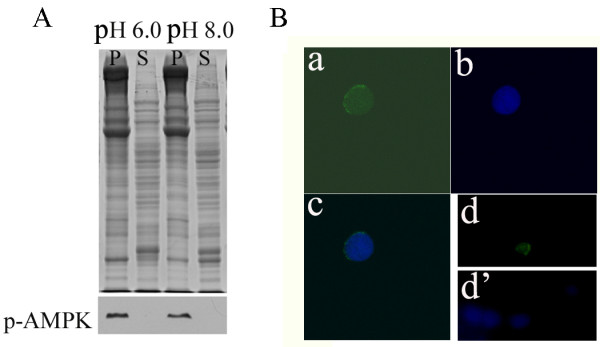
**Subcellular location of activated AMPK**. A. Supernatant (S) and pellet (P) fractions were prepared using buffer K (pH 6.0 or 8.0) from 4-h incubated embryos and separated by 10% SDS-PAGE. Proteins were also transferred to PVDF membranes and detected using phospho-AMPK (Thr172) antibody. The detected AMPK is indicated by an arrow. B. Confocal microscopy confirms that AMPK localizes in the nuclei when activated. Nuclei were double-stained using DAPI (a) and FITC-conjugated goat anti-rabbit IgG (b) after incubation with phospho-AMPK (Thr172) antibody. c, merged image of a and b. d, d' low magnification image of a field of nuclei double-stained with FITC-conjugated goat anti-rabbit IgG (d) and DAPI (d').

The subcellular location of activated AMPK is closely related to its function. Thus, we used a cell fraction system to separate pellet and supernatant proteins of embryos [22]. The pellet fraction loaded on SDS-PAGE contains nuclei and yolk platelets, while the supernatant contains cellular proteins. As shown in Figure 3A, activated AMPK was detectable on Western blots of pellet extracts, but not in supernatant extracts. Immunofluorescent staining of nuclei and confocal microscopy further confirmed that activated AMPK was localized in the nucleus (Figure 3B; Additional file 1).

### Activation of AMPK is not associated with the cellular AMP:ATP ratio

**Figure 4:**
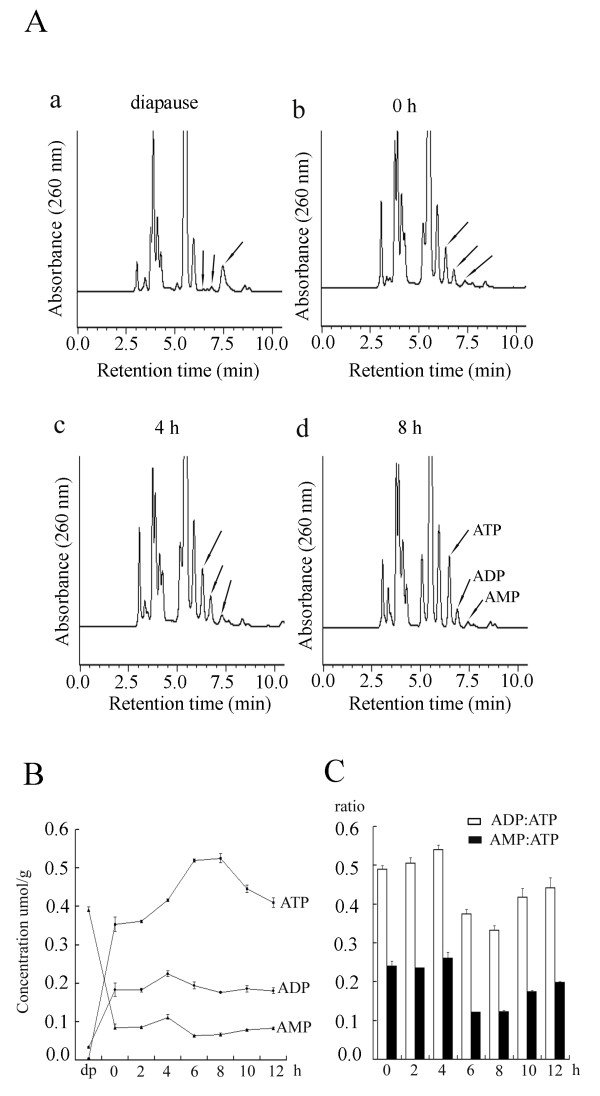
**Cellular ATP, ADP, and AMP were determined by HPLC analysis**. A. HPLC chromatogram of ATP, ADP, and AMP from *Artemia *diapause embryos (a) and embryos at three representative developing stages (0, 4, and 8 h, b-d). Arrows indicate peaks of ATP, ADP, and AMP. B. Concentrations of cellular ATP, ADP, and AMP in embryos at different developmental stages were determined by an external standard method. dp, diapause embryos. C. Changes of ADP:ATP (white columns) and AMP:ATP (black columns) ratios in developing embryos.

Cellular ATP, ADP, and AMP were separated by HPLC analysis in embryos at different developmental stages (Figure 4A). Concentrations of cellular adenylates were calculated by the external standard method (Additional file 2). The results showed that diapause embryos contained large amounts of AMP, while ATP and ADP were merely detectable (Figure 4A, a; Figure 4B, dp). In contrast, ATP remained at a high level in embryos during development (Figure 4B, 0–12 h). The level of ATP increased from initiation of the development until it reached its maximal value at the 8th hour of development (Figure 4B, ATP, 0–8 h). The ATP concentration decreased in the next 4 hours, but still remained higher than at the initiation of development (Figure 4B, ATP, 8–12 h). During development, the AMP:ATP ratios were between 0.1 and 0.3 (Figure 4C). Interestingly, AMPK was not activated in the first 4 hours in which the AMP:ATP ratio was higher than the later stages.

## Discussion

AMPK is a family of serine/threonine protein kinases playing a central role in energy control, at both the cellular level and the organism as a whole [23]. Genes encoding the subunits of the AMPK complex have been identified in all eukaryotes in whom genomes have been sequenced, covering all taxa from yeast to mammals [16]. Herein we showed that *Artemia *AMPK is specifically activated during the post-diapause developmental stages, and that the spatial and subcellular location of activated AMPK provides valuable information for further investigation of its functions.

Post-diapause development of *Artemia *is a very complicated process involving a large number of internal events, including RNA and protein synthesis restoration, cellular differentiation, and associated morphologic changes [11,12,16,24,25]. All these biochemical reactions occur together with large energy fluctuations. Our Western blotting results showed that AMPKα is specifically activated during this process, but not in other stages of the *Artemia *life cycle (Figure 1A). AMPK is a central component of a protein kinase cascade that plays a pivotal role in the regulation of the intracellular energy status. It has been established that activation of AMPK is associated with increased expression of key metabolic proteins, such as hexokinase II, GLUT-4, and mitochondrial enzymes [23,26]. Thus, *Artemia *AMPK may be a potential candidate for an energy control function during the post-diapause developmental stages. However, the fact that *Artemia *AMPK activation was not detected before the 4th hour of development indicates that AMPK functions may be development-related.

As a downstream target of LKB1, AMPK was shown to be indispensable in embryogenesis. It has been found that AMPKα-null mutants of *Drosophila *die before adulthood and are unable to reach the larval stage when both zygotic and maternal AMPK contributions are eliminated [27]. In *C. elegans*, orthologues of LKB1 (par-4) and AMPK (aak1, aak2) cooperate to regulate germline proliferation with somatic development during dauer formation [28]. Thus, *Artemia *AMPK may not only function as an energy regulator, but also plays other roles during post-diapause developmental stages. To further explore the role of AMPK in this process, we also conducted a whole-mount immunohistochemistry experiment in developing cysts. Interestingly, our results show that activated AMPK is predominantly restricted in the ectoderm region of embryos and the position of the activation loop changes as development proceeds (Figure 2). Thus, AMPK is probably involved in the embryonic development process, such as differentiation of certain cells and morphologic changes.

Studies have shown that AMPK may regulate gene expression through direct interaction with the nucleus [15,16]. The observation that *Artemia *AMPK localizes to the nucleus when activated (Figure 3) suggests that *Artemia *AMPK interacts with transcriptional regulators or DNA directly to control gene expression. This finding, combined with the results that activated *Artemia *AMPK exists in certain cells and then its location changes during development (Figure 2), led to the hypothesis that AMPK may participate in regulating the expression of genes that function in development of certain organs or tissues.

Thus far, two proteins (p26 and p8) have been established as nuclear binders in *Artemia *[1,29]. p8 is the first diapauses-related transcription factor to be identified in crustaceans, while p26, making up 10–15% of the total non-yolk protein of *Artemia *diapause embryos, serves primarily as a cell stress chaperone [29,30]. Interestingly, nuclear-cytoplasmic translocations of p26 are pH-dependent. Additionally, previous work by Utterback and Hand [11] has shown that alteration of pHi influences yolk platelet degradation during post-diapause development in *Artemia *embryos. Thus, we also examined the pH-dependence of nuclear-cytoplasmic translocations of activated AMPK by manipulating buffer pH *in vitro *(Figure 3A). The results showed that the location of activated AMPK in the nucleus was not affected by pHi.

It is known that AMPK is activated by conditions leading to an increase in the intracellular AMP:ATP ratio [16]. However, our studies have shown that *Artemia *AMPK is not activated in diapause embryos in which the AMP concentration is rather high (Figure 4B), but is activated in developing cysts from the 4th hour of post-diapause developmental stages in which the intracellular AMP:ATP ratio is low (Figure 4C). This result seems uncoupled to traditional mechanisms of AMPK activation by an elevated AMP:ATP ratio [16,23]. During the post-diapause developmental stages, however, it was notable that AMPK was only activated in certain cells of developing cysts, as revealed by the whole-mount immunohistochemistry results (Figure 2). There are two possible reasons for the activation of AMPK in this process: 1) although the AMP:ATP ratio is low in whole embryos, in the cells in which AMPK is activated, the AMP:ATP ratio is high; if so, AMPK activation is consistent with the classic regulatary mechanisms by the AMP:ATP ratio, and 2) although the AMP:ATP ratio is a major signal for AMPK activation, *Artemia *AMPK could also be regulated by other pathways which are independent of the cellular adenosine nucleotide level. This type of AMPK activation regulation by other cell signals has been reported in other organisms. In human cells, AMPK activity is regulated by two upstream kinases, LKB1 and CaMKK [17,18]. Regulation of the AMPK activity by CaMMK is not affected by the AMP:ATP ratio.

As is known, classic AMPK activation stimulates ATP-producing, catabolic pathways and inhibits ATP-consuming anabolic pathways [23]. Thus, it could be hypothesized that AMPK activation in the whole embryo during the post-diapause development process would inhibit ATP-consuming processes, such as protein synthesis and cell growth. However, it is notable that AMPK was only activated in certain cells (Figure 2). This fact indicates that even if *Artemia *AMPK is activated to switch off the ATP-consuming pathways in some cells, it would probably not inhibit the anabolic activity of the whole embryo. Furthermore, the fact that AMPK enters nuclei suggests that AMPK may play more roles in regulating the expression of the development-related genes rather than directly controlling cell energy. Considering the inhibition of anabolic metabolism by AMPK, it can also be hypothesized that the stimulation of AMPK contributes to metabolic depression and the inhibition of proliferation in the diapause embryos in which the AMP level is high and the metabolic activity is undetectable. However, AMPK was not detectably activated in the diapause state (Figure 1A). One possible reason is that AMPK is not the key factor that controls or maintains diapause. Thus, in the diapause state, as with other key metabolic enzymes, such as S6 kinase and hexokinase[25,31], AMPK loses its activity in spite of high AMP concentrations.

## Conclusion

In summary, our study focused on AMPK activation during the *Artemia *life cycle. The spatial-temporal activation pattern of AMPK, as well as its subcellular location, helped to further investigate its functions in *Artemia *post-diapause developmental stages. Our findings may thus provide insight into the regulation of energy and gene expression during *Artemia *post-diapause development, and reveal further aspects of AMPK function.

## Methods

### Culturing of Brine Shrimps and Embryos for Sampling

Post-diapause embryos of *Artemia franciscana*, gifted by Freshwater Fisheries Institute of Zhejiang (China), were incubated in ice-cold artificial sea water (3%) under continuous light at 25°C following hydratation at 4°C for 5 hours. Sampling was done at 0, 2, 4, 6, 8, 10, and 12 h, and at the free-swimming nauplius stage. *Artemia *were kept at room temperature (25–27°C) and fed once a day with *Chlorella *powder. Encysted embryos were gathered and stored dry at 25°C as a sample set of diapause embryos and used within 2 weeks. All samples were used immediately or snap-frozen in liquid nitrogen and stored at -80°C until use.

### Western Blotting on Whole Protein Extracts

Proteins were extracted from each sample using the TRIZOL reagent (Invitrogen, Carlsbad, CA, USA) according to the manufacturer's instructions, and were quantified using the Bradford method [32]. Fifty μg of protein of each sample were separated on 10% SDS-polyacrylamide gels (SDS-PAGE) and transferred to PVDF membranes (Millipore, Bedford, MA, USA). The membranes were incubated with phospho-AMPK (Thr172) (1:1000; Cell Signaling Technology, Beverly, MA, USA) and α-tubulin (1:1000; Beyotime, Shanghai, China) antibodies overnight at 4°C, and the detection was performed using the BM Chemiluminescence Western Blotting Kit (Roche, Mannheim, Baden-Wurttenberg, Germany).

### Expression of GST-SAMS

For the expression of GST-SAMS, a recombinant plasmid was constructed. In brief, two oligos (5'-GATCCCATATGCGCAGCGCGATGAGCGGCCTGCATCTGGTGAAACGCCGCTGAC-3' and 5'-TCGAGTCAGCGGCGTTTCACCAGATGCAGGCCGCTCATCGCGCTGCGCATATGG-3', 10 μM each) were mixed together in TN buffer (10 mM Tris [pH 8.0] and 10 mM NaCl), heated at 70°C for 5 min, and then cooled down slowly to room temperature. The annealed product was then subcloned in pGEX4T1 (Amersham Biosciences) at BamHI and XhoI sites. The nucleotide sequence of the recombinant plasmid was confirmed by DNA sequencing. The GST-SAMS or GST was expressed in BL21(DE3)pLysS of *E. coli *strain using IPTG induction (0.1 mM) at 20°C overnight. GST-SAMS or GST from 5 ml of cell culture was bound to 50 μl of glutathione beads using Glutathione Sepharose™ 4B (GE Healthcare, Piscataway, NJ).

### In vitro kinase assay

Embryos were homogenized in a homogenization buffer (20 mM Tris [pH 7.5], 150 mM NaCl, 1 mM EDTA, 1 mM EGTA, 250 mM mannitol, 50 mM sodium fluoride, 1% Triton X-100, 2.5 mM sodium pyrophosphate, 1 mM b-glycerophosphate, 1 mM Na3Vo4, and 1 ug/ml leupeptin). The homogenate was centrifuged and the supernatant was subjected to polyethylene glycol (PEG) precipitation.

AMPK activity was measured using the GST-SAMS phosphorylation assay. The kinase assay was performed at 30°C for 20 min with 100 μg of PEG extracts in assay buffer containing 40 mM HEPES, 0.2 mM AMP, 80 mM NaCl, 8% glycerol, 0.8 mM DTT, 5 mM MgCl_2_, and 0.2 mM ATP with 5 ul of GST-SAMS or GST binding beads. After reaction, the beads were quickly centrifuged and washed with TBS buffer. Then, the beads were boiled in 2× protein loading buffer for 5 min and centrifuged. The supernatants were separated by 15% SDS-PAGE, and phosphorylated proteins were detected by Western blotting using Biotinylated Phos-tag™.

### Immunohistochemistry on whole mount embryos

For whole mount immunohistochemical studies, cysts were decapsulated using antiformin [22]. Decapsulated embryos were fixed in 4% paraformaldehyde, and permeated by 0.1% Triton X-100. Phospho-AMPK (Thr172) antibody (1:100) was used as the primary antibody. The secondary antibody was AP-conjugated goat anti-rabbit IgG secondary antibody (1:350; Promega, Madison, WI, USA). The staining was performed using the NBT/BCIP solution in the dark.

### Nuclear translocation of AMPK

Cell fractionation was performed as described [22]. Briefly, 4-h embryos were homogenized using Dounce homogenizers in buffer K (pH 6.0 or 8.0). Homogenates were centrifuged at 1630 g for 5 min at 2°C to obtain supernatant and pellet fractions. Pellets (nuclei, yolk platelets, and shell fragments) were washed once with 200 times volume of buffer K and restored to initial volumes. After being heated in 2× sample buffer at 100°C for 5 min, insoluble shell fragments were removed by centrifugation at 1630 g for 5 min [33]. Supernatants were electrophoresed in 10% SDS-PAGE, and proteins were detected by Coomassie blue-G staining. Proteins from SDS-PAGE were also transferred to PVDF membranes and incubated with Phospho-AMPK (Thr172) antibody as described above.

### Immunofluorescent Staining of Nuclei

Nuclei of 6-h embryos were prepared by centrifugation on Percoll gradients, fixed in 4% (w/v) paraformaldehyde, and processed as described [3]. Phospho-AMPK (Thr172) antibody (1:100) was used as the primary antibody. The secondary antibody was FITC-conjugated goat anti-rabbit IgG (1:100; Sigma, St. Louis, MO, USA). After a 30-min incubation with secondary antibody, the nuclei were rinsed with PBS, and incubated with DAPI (Beyotime, Shanghai, China). Slides were examined with either a Nikon ECLIPSE TE200-S microscope with an argon/krypton laser or a Zeiss LSM 510 inverted laser scanning confocal microscope.

### Determination of ATP, ADP, and AMP Concentrations

Diapause embryos and post-diapause embryos that developed for 0, 2, 4, 6, 8, 10, and 12 h were collected and rinsed by sterile water. Each sample contained at least 20 mg of embryos. Excessive water was removed before adenylate (ATP, ADP, and AMP) extraction with perchloric acid [34]. The HPLC conditions were as follows: a SHIMADZU CLC-C8 250*4.60 mm column was equipped with a SHIMADZU LC-10AT pump system. Mobile phase A consisted of 0.18 M K_2_HPO_4 _and 0.1 M KH_2_PO_4 _dissolved in deionized water, while mobile phase B consisted of 65% acetonitrile. The flow rate of the mobile phase was 1 ml/min, while the injection volume was 10 μl. Peaks were detected and analyzed at 260 nm, and the column temperature was 30°C. The elution program was as follows: 0 min 100% A, 0% B; 10 min 100% A, 0% B; 18 min 0% A, 100% B; 23 min 0% A, 100% B; 25 min 100% A, 0% B; and 35 min stop. ATP, ADP, and AMP were identified by comparison with retention time of standards (Sangon, Shanghai, China) while their concentrations were determined using the external standard method. Data was expressed as the means ± s.e.m. of three replicate determinations.

## Authors' contributions

XJZ designed and performed the experiments, and wrote the paper. JQD participated in the Western blotting analysis and the whole mount immunohistochemistry. XT participated in the determination of adenylate concentrations. YZ participated in the kinase activity assay. WJY supervised and participated in the design of the study. All authors read and approved the final manuscript.

## Supplementary Material

Additional file 1 Phosphorylated AMPK is found in different layers of nuclei (a-o) from 4-h incubated embryos by immunofluorescent staining of nuclei and confocal microscopy. The data provide confocal microscopy images of sections of a single nucleus double-stained with DAPI and FITC-conjugated goat anti-rabbit IgG after incubation with phospho-AMPK (Thr172) antibody. Click here for file

Additional file 2 Concentrations of cellular ATP, ADP, and AMP in embryos at different developmental stages (0–12 h; μm per 1 g wet weight embryos). The data provided represent the statistical analysis of cellular adenylates concentration in various embryos. Click here for file
