# Supplementary figures and images for: Activation of an AMP-activated protein kinase is involved in post-diapause development of Artemia franciscana encysted embryos
Source: BMC Dev Biol. 2009 Mar 16;9:21. doi: 10.1186/1471-213X-9-21 (PMC2667496; doi:10.1186/1471-213X-9-21)

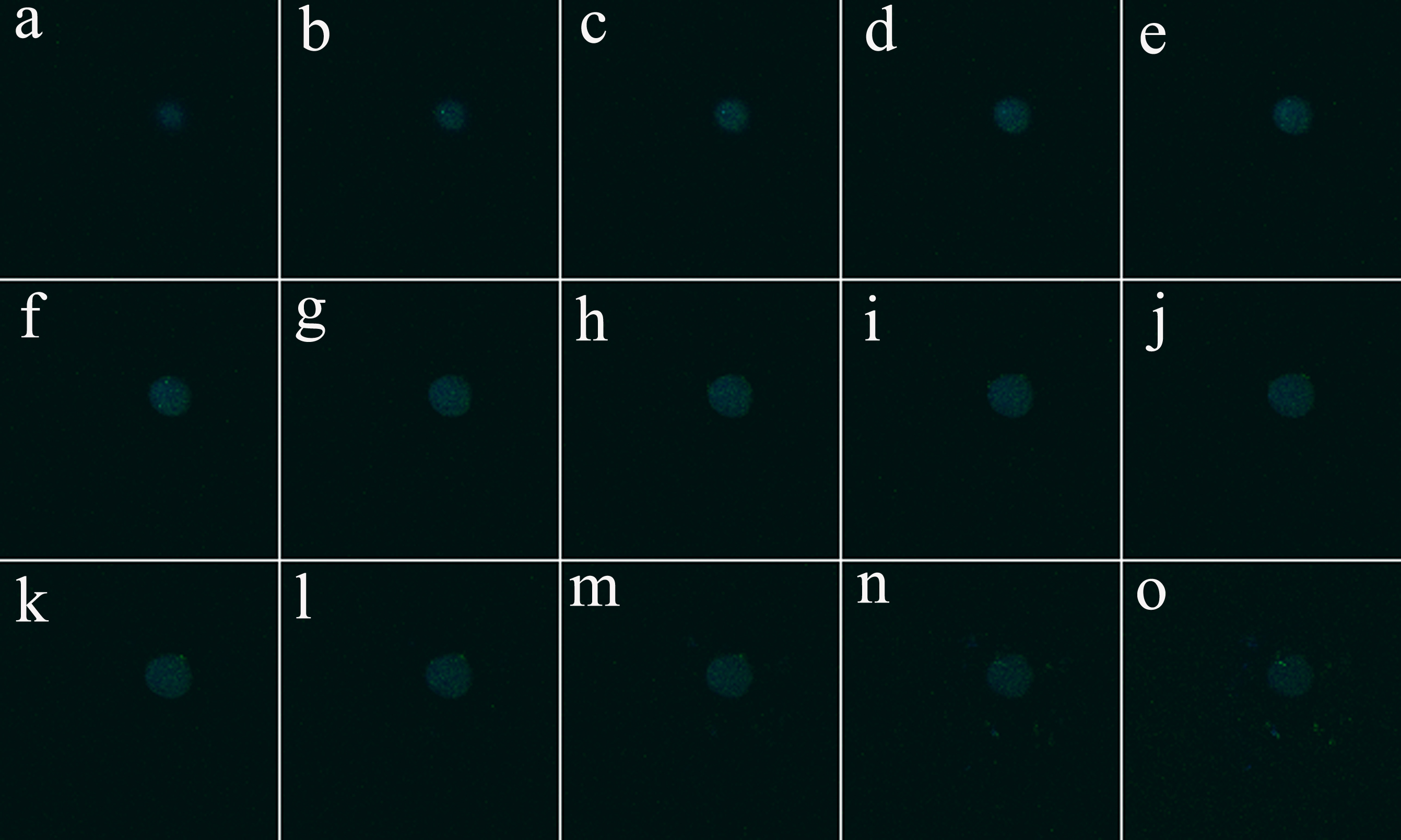

Supplement: Additional file 1 — Phosphorylated AMPK is found in different layers of nuclei (a-o) from 4-h incubated embryos by immunofluorescent staining of nuclei and confocal microscopy. The data provide confocal microscopy images of sections of a single nucleus double-stained with DAPI and FITC-conjugated goat anti-rabbit IgG after incubation with phospho-AMPK (Thr172) antibody. [file 1471-213X-9-21-S1.jpeg]
