# Supplementary material for: Activation of an AMP-activated protein kinase is involved in post-diapause development of Artemia franciscana encysted embryos
Source: BMC Dev Biol. 2009 Mar 16;9:21. doi: 10.1186/1471-213X-9-21 (PMC2667496; doi:10.1186/1471-213X-9-21)
Supplement: Additional file 2 — Concentrations of cellular ATP, ADP, and AMP in embryos at different developmental stages (0–12 h; μm per 1 g wet weight embryos). The data provided represent the statistical analysis of cellular adenylates concentration in various embryos. [file 1471-213X-9-21-S2.doc]

Additional file 2. Concentrations of cellular ATP, ADP, and AMP in embryos at different developmental stages (0-12 h; μm per 1 g wet weight embryos). dp, diapause embryos. Data are the means ± s.e.m. of three replicate determinations

|  | | ATP | ADP | AMP |
| --- | --- | --- | --- | --- |
| dp | | 0.0036±0.0006 | 0.0327±0.0015 | 0.3903±0.0093 |
| 0 h | | 0.3539±0.0185 | 0.1832±0.0170 | 0.0851±0.0087 |
| 2 h | | 0.3610±0.0039 | 0.1824±0.0044 | 0.0860±0.0007 |
| 4 h | | 0.4168±0.0035 | 0.2249±0.0074 | 0.1103±0.0086 |
| 6 h | | 0.5201±0.0041 | 0.1945±0.0093 | 0.0637±0.0007 |
| 8 h | | 0.5257±0.0115 | 0.1765±0.0025 | 0.0659±0.0044 |
| 10 h | 0.4462±0.0100 | | 0.1860±0.0085 | 0.0788±0.0010 |
| 12 h | 0.4103±0.0127 | | 0.1806±0.0080 | 0.0827±0.0035 |
